# Would initiating colorectal cancer screening from age of 45 be cost-effective in Germany? An individual-level simulation analysis

**DOI:** 10.3389/fpubh.2024.1307427

**Published:** 2024-02-21

**Authors:** Min Wai Lwin, Chih-Yuan Cheng, Silvia Calderazzo, Christoph Schramm, Michael Schlander

**Affiliations:** ^1^Division of Health Economics, German Cancer Research Center (DKFZ), Heidelberg, Germany; ^2^Medical Faculty Mannheim, University of Heidelberg, Mannheim, Germany; ^3^Division of Biostatistics, German Cancer Research Center (DKFZ), Heidelberg, Germany; ^4^Clinics of Gastroenterology, Hepatology and Transplantation Medicine, Essen University Hospital, Essen, Germany

**Keywords:** cancer screening, colorectal cancer, cost-effectiveness, CRC, discrete event simulation, early-onset CRC, modeling

## Abstract

**Background:**

Colorectal cancer (CRC) screening has been shown to be effective and cost-saving. However, the trend of rising incidence of early-onset CRC challenges the current national screening program solely for people ≥50 years in Germany, where extending the screening to those 45–49 years might be justified. This study aims to evaluate the cost-effectiveness of CRC screening strategies starting at 45 years in Germany.

**Method:**

DECAS, an individual-level simulation model accounting for both adenoma and serrated pathways of CRC development and validated with German CRC epidemiology and screening effects, was used for the cost-effectiveness analysis. Four CRC screening strategies starting at age 45, including 10-yearly colonoscopy (COL), annual/biennial fecal immunochemical test (FIT), or the combination of the two, were compared with the current screening offer starting at age 50 years in Germany. Three adherence scenarios were considered: perfect adherence, current adherence, and high screening adherence. For each strategy, a cohort of 100,000 individuals with average CRC risk was simulated from age 20 until 90 or death. Outcomes included CRC cases averted, prevented death, quality-adjusted life-years gained (QALYG), and total incremental costs considering both CRC treatment and screening costs. A 3% discount rate was applied and costs were in 2023 Euro.

**Result:**

Initiating 10-yearly colonoscopy-only or combined FIT + COL strategies at age 45 resulted in incremental gains of 7–28 QALYs with incremental costs of €28,360–€71,759 per 1,000 individuals, compared to the current strategy. The ICER varied from €1,029 to €9,763 per QALYG, and the additional number needed for colonoscopy ranged from 129 to 885 per 1,000 individuals. Among the alternatives, a three times colonoscopy strategy starting at 45 years of age proves to be the most effective, while the FIT-only strategy was dominated by the currently implemented strategy. The findings remained consistent across probabilistic sensitivity analyses.

**Conclusion:**

The cost-effectiveness findings support initiating CRC screening at age 45 with either colonoscopy alone or combined with FIT, demonstrating substantial gains in quality-adjusted life-years with a modest increase in costs. Our findings emphasize the importance of implementing CRC screening 5 years earlier than the current practice to achieve more significant health and economic benefits.

## 1 Introduction

Colorectal cancer (CRC) ranks third globally in prevalence and second in cancer mortality, with 1.9 million new cases and 0.9 million deaths reported in 2020. These numbers are projected to increase to 3 million new cases and 1.6 million deaths annually by 2040 (1). The total annual cost of CRC in Europe in 2015 was estimated at €19.1 billion (2), and this economic burden is expected to rise as the population ages and the incidence of CRC increases. Furthermore, over the last decade, there has been a growing trend of early-onset CRC among individuals under the age of 50 (3, 4).

Given the high incidence and low survival rates of CRC in advanced stages, prevention and early detection of CRC has been recognized as a critical approach (5–7). Polyps are the precursors to most cases of CRC and typically take over a decade to progress to carcinoma (8, 9). These precancerous lesions can be detected early through screening and removed, making prevention a viable strategy (6, 10). Between 2008 and 2018, colorectal cancer incidence showed a decreasing trend in some EU countries where population screening programs are in place, suggesting the effectiveness of screening programs (11).

In 2002, Germany introduced the CRC screening covered by Statutory Health Insurance (SHI) for people aged over 50 (12). In April 2019, the nationwide organized CRC screening program was launched, offering colonoscopy and FIT as screening test options to eligible individuals who receive personal invitation letters from SHI at ages 50, 55, 60, and 65 (see Table 1). The change from opportunistic to organized screening with invitations aimed to increase participation rates and hence screening effectiveness (14). Limited evidence on cost-effectiveness of CRC screening strategies in Germany has posed challenges for policy decision-making.

**Table 1:** Current colorectal cancer screening strategy in Germany.

	Screening test	
	Colonoscopy	FIT^a^
Men	Starting eligible age: 50 years old Entitled to 2 screening colonoscopies if the first was done before the age of 65	If no screening colonoscopy is used -age 50–54 years: annually -age ≥55 years: biannually
Women	Starting eligible age: 55 years old Entitled to 2 screening colonoscopies if the first was done before the age of 65	-age 50–54 years: annually If no screening colonoscopy is used -age ≥55 years: biannually

FIT, fecal immunochemical test.

a FIT positive individuals will receive a follow-up colonoscopy. Reference: (13).

A cost-effectiveness analysis (CEA) study conducted in 2014 using a Markov model approach provided insights during the period of opportunistic screening with guaiac fecal occult blood test (gFOBT)/fecal immunochemical test (FIT) and colonoscopy (15). However, no CEA to date has been conducted in Germany to address the rising incidence of early-onset colorectal cancer, along with the introduction of organized screening programs.

The objectives of this study are to evaluate the cost-effectiveness of initiating CRC screening at age 45 versus age 50 and provide evidence-based recommendations for optimizing the current screening guidelines.

## 2 Method

### 2.1 Modeling approach

In this study, DECAS (Discrete Event simulation model for the natural history of colorectal cancer from the Adenoma and Serrated neoplasia pathways) was used to simulate the long-term outcomes of alternative CRC screening strategies. DECAS is the first individual-level CRC screening model simulating the natural history of CRC progression from both adenoma and serrated pathways and calibrated using a Bayesian method (16–18). The model considers differences in dwell time and rate between the two pathways. Details about DECAS model structure, assumptions, calibration and validation for both natural history and screening effects were published elsewhere (19). To illustrate the main structure of DECAS model, we have provided a schematic diagram and the CRC natural history parameters in the Supplementary Figure S1; Supplementary Table S1.

For each CRC screening strategy, a cohort of 100,000 average-risk individuals without prior screening or CRC diagnosis were followed from age 20 to 90 or until death. Each cohort was simulated 1,000 times using random posterior parameters obtained from the Bayesian calibration during DECAS development, and the average outputs from these simulations were reported. This study took a healthcare system perspective.

### 2.2 Screening strategies for comparison

Strategy 1 represents the current CRC screening offers in Germany. We designed four new screening strategies at age 45 to evaluate their cost-effectiveness relative to the current one. Strategy 2, termed FIT1y45 + COL10y50, entails annual FIT from 45 to 49 years of age followed by colonoscopies at ages 50 and 60. Strategy 3, FIT1y45 + COL10y50-3X, include annual FIT from 45 to 49 years but extends the colonoscopy schedule to ages 50, 60, and 70. Strategy 4, FIT2y45, denotes biennial FIT from 45 to 75 years. Strategy 5, COL10y45-3X strategy involves three colonoscopies at ages 45, 55, and 65, with no FIT component. These strategies involve different combination of FIT and colonoscopy, commencing screening at age 45, offering diverse test options and timing (20). Refer to Table 2 for detailed information on these screening strategies.

**Table 2:** Overview of screening strategies for comparison.

No.	Abbreviation	Screening strategy description
0	No screening	No CRC screening in lifetime
1	mCOL50/fFIT55 + COL55 (current strategy as comparator)	Men: 2 colonoscopies at age 50 and 60 years;
		Women: annual FIT for age 50–54 years followed by 2 colonoscopies at 55 and 65 years
2	FIT1y45 + COL10y50	Annual FIT for age 45–49 years followed by 2 colonoscopies at 50 and 60 years
3	FIT1y45 + COL10y50-3X	Annual FIT for age 45–49 years followed by 3 colonoscopies at 50, 60 and 70 years
4	FIT2y45	Biennial FIT for age 45–75 years
5	COL10y45-3X	3 colonoscopies at age 45, 55 and 65 years

COL, colonoscopy; FIT, fecal immunochemical test.

### 2.3 Scenarios of screening participation

The level of participation (adherence) plays a critical role in determining the effectiveness of population based CRC screening programs. Our study examined three different scenarios: (1) perfect adherence, (2) current observed adherence in Germany, and (3) high participation rates observed in selected European programs. See Table 3 for detailed adherence scenarios.

**Table 3:** Summary of three scenarios with different screening participation.

Test options	Participation rate (%)		
	1. Perfect adherence	2. Current program	3. High adherence
Annual FIT	100%	Men 7%, women 25%	Men 71%, women 75%
Biennial FIT	100%	Men 16%, women 24%	Men 71%, women 75%
FIT-positive COL	100%	64%	83%
10-yearly screening COL	100%	Men 23%, women 24%	42%
Surveillance COL	100%	63%	63%

COL, colonoscopy; FIT, fecal immunochemical test. Reference: (21–24).

Scenario 1 assumes perfect participation and follow-up, which represents the highest potential effect of the population screening program. Scenario 2 reflects estimated current adherence rates in Germany based on existing literature for screening, follow-up, and surveillance rates. Based on German Federal Office of Statistics data, screening participation rates in pre-organized program were 7% (male) and 25% (female) in annual FIT screening, 16% (male) and 24% (female) in biennial FIT screening, and 17% (male) and 19% (female) in 10-yearly colonoscopy screening. Studies suggest organized programs and invitations moderately impact FIT participation but could increase colonoscopy participation by 1.3 times (21, 25). Thus, we assumed 10-yearly colonoscopy rates at 23% (male) and 24% (female), with FIT-positive colonoscopy adherence at 64% per a German study (25).

Lastly, a higher adherence scenario (Scenario 3) is formulated, inspired by successful European CRC screening programs like those in the Netherlands and Basque country (Spain). These programs achieve >70% participation rates by sending advanced notifications before FIT kit mailing, along with reminders 4–6 weeks later (26). We adopted these strategies, anticipating a strong uptake of 71% (male) and 75% (female) for FIT, and 83% for FIT-positive colonoscopy, aligned with the Dutch program (26). With additional reminder letters, 10-yearly colonoscopy uptake was estimated at 42% for both genders, based on US randomized studies (22).

Screening follow-up and surveillance management after colonoscopy, lesion removal and biopsy procedures are scheduled accordingly with the German S3 guidelines for CRC follow-up colonoscopy. Please see detailed information in the Supplementary Table S2 for assumptions used in the DECAS model for the surveillance colonoscopy intervals.

### 2.4 Model input parameters for screening interventions

All model inputs are summarized in Table 4. The sensitivities of colonoscopy were referenced from two meta-analyses that assessed miss rates for adenomas, serrated lesions, and CRC in the screening context (30, 37). The sensitivity and specificity of the FIT were based on values obtained from a meta-analysis specifically focusing on FIT test sensitivities at a threshold of 20 μg hemoglobin/g of stool (27).

**Table 4:** Summary of model inputs and values for probabilistic sensitivity analysis for the cost-effectiveness analysis of German colorectal cancer screening program.

	Input value		PSA		Reference
	Mean	95% CI	Distribution	Range	
Screening test performance					
FIT					
Sensitivity					
Non-AA	0.08	0.07–0.09	Uniform	0.07–0.09	(27–29)
AA	0.26	0.2–0.32	Uniform	0.2–0.32	
Non-crSP	0.07	0.03–0.15	Uniform	0.03–0.15	
crSP	0.11	0.04–0.25	Uniform	0.04–0.25	
Cancer	0.77	0.66–0.85	Uniform	0.66–0.85	
Specificity	0.95	0.92–0.96	Uniform	0.92–0.96	
Colonoscopy					
Sensitivity					
Non-AA	0.76	0.7–0.77	Uniform	0.7–0.77	(20, 30)
AA	0.91	0.84–0.96	Uniform	0.84–0.96	
Non-crSP	0.73	0.6–0.84	Uniform	0.6–0.84	
crSP	0.76	0.63–0.87	Uniform	0.63–0.87	
Cancer	0.95	0.86–1	Uniform	0.86–1	
Specificity	1	–	Uniform	–	
Screening complications					
Major bleeding & perforation from colonoscopy	0.0004	–	Uniform	0.0002–0.0024	(31, 32)
Utility					
Baseline	0.85	0.83–0.88	Uniform	0.83–0.88	(33)
CRC stage 1–4, initial phase	0.76	0.7–0.82	Uniform	0.7–0.82	
CRC stage 1–3, continuing phase	0.84	0.78–0.88	Uniform	0.78–0.88	
CRC stage 4, continuing phase	0.82	0.78–0.86	Uniform	0.78–0.86	
CRC stage 1–4, terminal phase	0.64	0.55–0.75	Uniform	0.55–0.75	
Utility loss (per event)					
Due to colonoscopy itself	0.0005	–	Uniform	0.0004–0.0006	(20)
Due to waiting for FIT results	0.0013	–	Uniform	0.0010–0.0016	
Due to waiting for polypectomy results	0.0009	–	Uniform	0.0007–0.0011	
Due to colonoscopy complications	0.0055	–	Uniform	0.0044–0.0066	
Costs (2023 Euro)					
Screening related					
Posting notification/reminders	€ 0.85	–	–	–	Assumption
Screening consultation (one-off)	€ 13.41	–	–	–	Assumption
FIT kit	€ 8.67	–	–	–	(34)
FIT process & analysis	€ 6.59	–	–	–	
Colonoscopy	€ 204.13	–	–	–	
Colonoscopy + polypectomy	€ 234.08	–	–	–	
Pathology test	€ 15.15	–	–	–	
Treatment for colonoscopy complication	€ 5,170	–	Uniform	5,117-5,299	(35)
Treatment for CRC					
Stage 1 & 2					(36)
Initial phase	€ 16,597	14,433–18,761	Uniform	14,433–18,761	
Continuing phase	-€ 1,006	1,263–645	Uniform	1,263–645	
Terminal phase	€ 31,007	23,406–38,610	Uniform	23,406–38,610	
Stage 3					
Initial phase	€ 38,085	34,688–41,480	Uniform	34,688–41,480	
Continuing phase	€ 2,038	918–3,156	Uniform	918–3,156	
Terminal phase	€ 24,266	19,719–28,812	Uniform	19,719–28,812	
Stage4					
Initial phase	€ 64,187	58,185–70,187	Uniform	58,185–70,187	
Continuing phase	€ 14,657	12,042–17,275	Uniform	12,042–17,275	
Terminal phase	€ 34,206	29,089–39,324	Uniform	29,089–39,324	

AA, advanced adenoma; CI, confidence interval; CRC, colorectal cancer; crSP, clinically relevant serrated polyps; FIT, fecal immunochemical test; PSA, probabilistic sensitivity analysis.

Various screening-related costs relevant to the German healthcare system were considered, including expenses associated with sending invitation letters and test kits, conducting screening consultations, performing colonoscopies, and addressing possible complications. Additionally, cancer treatment costs were determined using data from a previous study that examined claimed database records from a German SHI system, analyzing annual colon cancer treatment costs according to cancer severity and phase (initial, continuing, and terminal phases) (36). All costs were adjusted to 2023 Euro values using the Health Consumer Price Index specific to Germany (38).

As specific utility data corresponding to CRC disease states in the German context were unavailable, utility values were sourced from a Finnish study. The study employed the European Quality of Life 5 Dimensions 3 Level Version (EQ-5D-3L) instrument to survey patients with local or advanced CRC across various treatment phases, including primary treatment, rehabilitation, remission, or palliative care (33). Moreover, DECAS model accounted for utility losses related to screening, encompassing discomfort and complications arising from screening colonoscopy, as well as anxiety experienced during the waiting period for screening test results (including FIT and biopsy after polypectomy) (20).

### 2.5 Model outcomes, cost-effectiveness analysis, and burden benefit analysis

The model results were obtained by aggregating data over the entire lifetime of each individual and reported per 1,000 40 years-old individuals. The screening benefit was measured by reductions in CRC incidence and mortality, quality-adjusted life-years gained (QALYG) and associated costs, compared to no screening. All costs and health outcomes were discounted from the age of 40, applying a base-case annual rate of 3% (20, 39).

Efficiency frontier analysis was utilized to identify the most efficient strategies in terms of cost-effectiveness (39). Incremental cost-effectiveness ratios (ICERs) were then calculated to compare alternative screening strategies against the current strategy. ICERs were determined by dividing the incremental discounted cost by the incremental discounted quality-adjusted life-years (QALYs) between the strategies (40). Additionally, the number needed to colonoscopy for each alternative strategy was considered as an important factor in practical implementation.

### 2.6 Sensitivity analyses

#### 2.6.1 Probabilistic sensitivity analyses

Given the nature of the DECAS model, which utilizes 1,000 sets of posterior parameters from Bayesian calibration in each simulation, probabilistic sensitivity analyses (PSA) are inherently included in the outputs. This applied to the CRC natural history parameters which were calibrated (19). To complete the PSA, ranges were specified for the remaining model inputs (such as test characteristics, complication rates, treatment costs, and utility values), and 1,000 random numbers were drawn from a uniform distribution within each range. Screening costs were the only inputs that remained unchanged. See Table 4.

#### 2.6.2 Cost-effectiveness acceptability curves

The net health benefit (NHB) method was employed to transform outcomes into units of health benefit (QALYs) for comparison. By comparing NHBs across different strategies at different willingness-to-pay (WTP) thresholds, the strategy with the highest NHB was considered the most cost-effective (40). Cost-Effectiveness Acceptability Curves (CEACs) utilize all simulated outputs to determine the probability of an intervention being cost-effective compared to alternatives at various WTP thresholds, ranging from €0 to €100,000.

#### 2.6.3 Monte Carlo simulation on the ICER

To appraise the cost-effectiveness of the optimal strategy among all (COL10y45-3X), a Monte Carlo simulation was performed using 1,000 random samples within the 95% confidence interval (CI) of mean incremental cost and QALYG. These values were visualized on the cost-effectiveness plane, addressing uncertainty within the respective confidence intervals.

## 3 Result

### 3.1 Effectiveness of screening initiating at age 45 on CRC incidence and mortality rates

All the CRC screening strategies in this analysis outperformed the no screening condition. The CRC screening strategies starting at 45 years of age, except for the FIT only strategy, could effectively prevent more CRC cases and deaths compared with the current screening strategy which start at 50 years of age. Assuming perfect adherence, FIT1y45 + COL10y50, FIT1y45 + COL10y50-3X, and COL10y45-3X strategies could result in a reduction of incidence by 1.18, 2.25, and 5.68 cases and mortality by 0.78, 1.18, and 2.03 cases per 1,000 individuals, respectively. In scenario 2 and 3 where the adherence is not perfect, the preventive effects on incidence and mortality still followed but to a lesser degree compared with the perfect adherence scenario. The FIT only strategy was dominated by the current practice strategy in all scenarios. More detailed results are presented in Table 5 and Supplementary Table S3.

**Table 5:** Modeled benefits and costs of strategies per 1,000 40 years-old individuals.

Strategy	CRC incidence	Incidence reduction^‡^	CRC mortality	Mortality reduction^‡^	dQALYs	dQALYG^‡^	dCost (€)	ΔdCost^‡^	ICER^‡^	ΔNNC^‡^
No Screening	57.92		27.05		19107.18		1,084,554			
Scenario 1 (perfect adherence)										
mCOL50/fFIT50 + COL55*	20.83	–	7.56	–	19177.80	–	754,393	–	–	–
FIT1y45 + COL10y50	19.65	1.18	6.78	0.78	19185.90	8.11	793,408	39,015	4,811	129
FIT1y45 + COL10y50-3X	18.58	2.25	6.37	1.18	19185.15	7.35	826,152	71,759	9,763	652
FIT2y45	35.77	−14.94	11.34	−3.78	19156.33	−21.47	847,507	93,114	Dominated	−1,363
COL10y45-3X	15.15	5.68	5.53	2.03	19205.36	27.56	782,753	28,360	1,029	885
Scenario 2 (current adherence)										
mCOL50/fFIT50 + COL55*	49.01	–	19.90	–	19134.57	–	895,412	–	–	–
FIT1y45 + COL10y50	48.46	0.56	19.67	0.23	19136.28	1.71	896,663	1,251	731	24
FIT1y45 + COL10y50-3X	47.74	1.28	19.42	0.48	19137.16	2.59	901,810	6,398	2,470	176
FIT2y45	55.65	−6.63	22.49	−2.58	19121.85	−12.72	948,385	52,973	Dominated	−411
COL10y45-3X	48.13	0.89	19.64	0.27	19139.77	5.20	906,330	10,918	2,100	197
Scenario 3 (high adherence)										
mCOL50/fFIT50 + COL55*	41.27	–	16.35	–	19145.96	–	883,574	–	–	–
FIT1y45 + COL10y50	38.57	2.70	14.99	1.36	19156.37	10.41	914,639	31,064	2,984	111
FIT1y45 + COL10y50-3X	37.47	3.80	14.58	1.77	19156.05	10.09	925,344	41,770	4,140	372
FIT2y45	45.88	−4.61	16.72	−0.37	19137.78	−8.18	1,032,285	148,710	Dominated	−440
COL10y45-3X	40.38	0.88	16.30	0.05	19155.10	9.13	875,290	−8,285	−907	311

*Current strategy, ‡ reference to current strategy, COL, colonoscopy; FIT, fecal immunochemical test; dQALYG, discounted quality-adjusted life-years gained. dCost, discounted lifetime cost; ΔdCost, discounted incremental cost; ICER, incremental cost effectiveness ratio; ΔNNC, number needed to colonoscopy. (1) The cost and quality-adjusted life-years were discounted with 3% annual rates. (2) Results are presented as mean values of 1,000 simulation of an age Cohort of 100,000 individuals (reported per 1,000 population). (3) Dominated denotes strategies being less effective and cost more.

### 3.2 Cost-effectiveness analysis: evaluating the cost and health benefits

**Figure 1:**
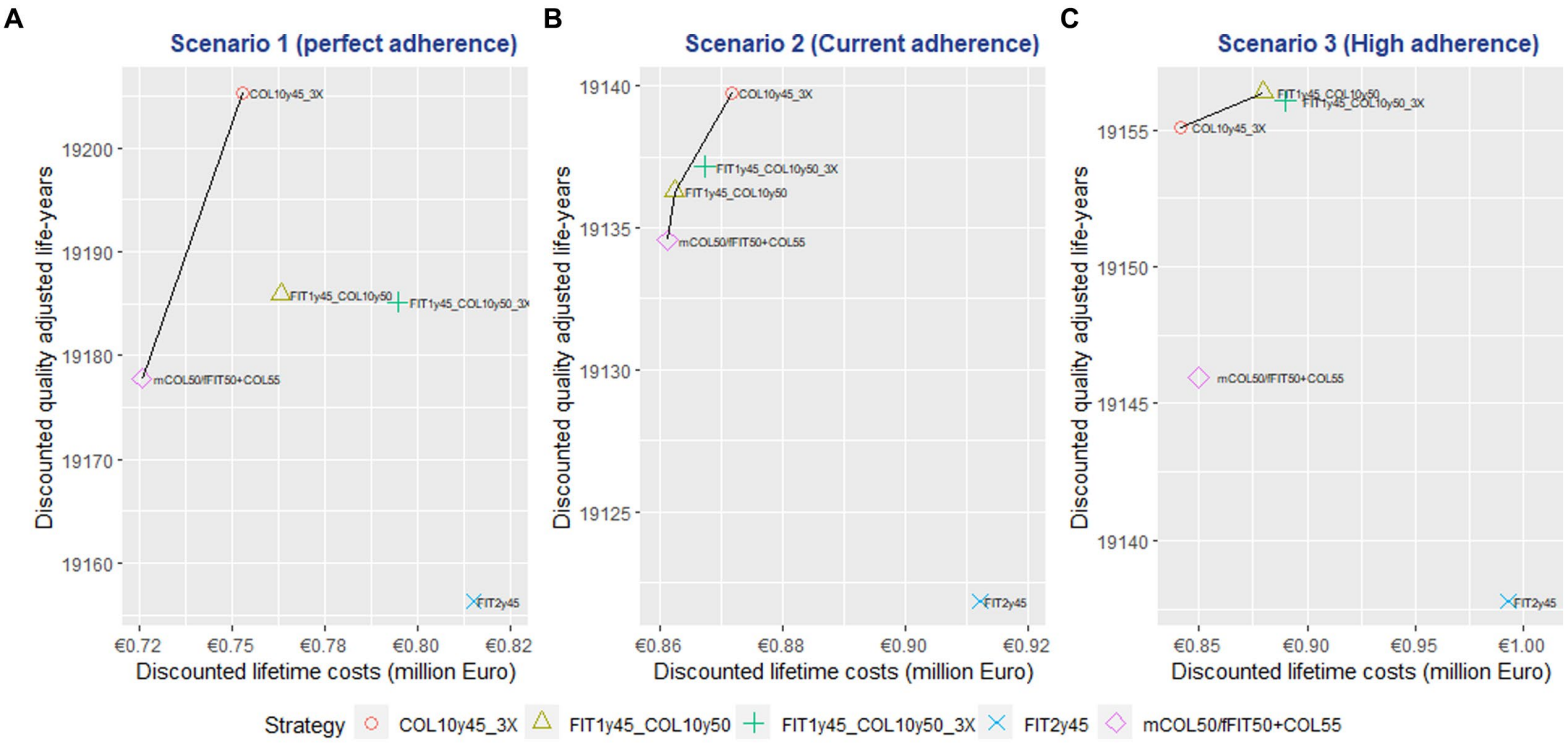
Cost-effectiveness plane of screening strategies in different adherence scenarios.

In scenario 1 (perfect adherence), the mCOL50/fFIT50 + COL55 and COL10y45-3X strategies were on the efficiency frontier of the cost effectiveness plane. Among the investigated strategies, the COL10y45-3X approach demonstrated superior performance, offering the highest QALY gained and with the smallest incremental cost. The mean ICER of this strategy was 1,029 € per QALY gained compared to the current strategy. However, it also required the highest incremental number of colonoscopies compared to the current strategy, with 885 per 1,000 individuals, due to being a colonoscopy-only strategy with three lifetime offers for each individual. See Table 5 and Figure 1.

In scenario 2 (current observed adherence rate in Germany), the mCOL50/fFIT50 + COL55, FIT1y45 + COL10y50, and COL10y45-3X strategies were on the efficiency frontiers. The FIT1y45 + COL10y50 strategy had the lowest ICER at 731 € per QALYG with 24 additional colonoscopy compared to the current strategy. Despite the COL10y45-3X strategy could deliver the highest QALY gained, it came with an incremental number of 197 colonoscopies per 1,000 population compared to the current strategy. See Table 5 and Figure 1.

In scenario 3 (high adherence), the COL10y45-3X and FIT1y45 + COL10y50 strategies were on the efficiency frontier. The COL10y45-3X resulted in lower cost and higher QALYs compared to the currently implementing strategy. This indicated that the strategy can not only improve health outcomes but also reduce costs. This outcome was advantageous from a health economics point of view. However, the combined strategies FIT1y45 + COL10y50 could provide higher QALY gained with some additional costs. The COL10y45-3X and FIT1y45 + COL10y50 strategies demanded an increase in the number of additional colonoscopies, a total of 311 and 111 respectively, when contrasted with the current strategy. See Table 5 and Figure 1.

### 3.3 Sensitivity analyses

**Figure 2:**
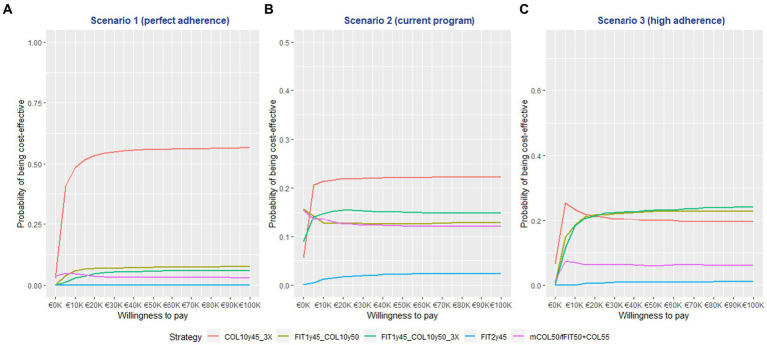
The cost effectiveness acceptability curve (CEAC) graph of the screening strategies at different willingness to pay thresholds.

The CEAC analysis demonstrated that the COL10y45-3X strategy had the highest probability of being cost-effective in the first two scenarios. This finding held true across a range of WTP thresholds, from €5,000 to €100,000 per QALYG. Notably, in Scenario 1, the COL10y45-3X strategy had a probability of over 50% for WTP > €15,000, emphasizing its cost-effectiveness based on QALY. See Figure 2 for detailed result. In scenario 3, the combined modality strategies (FIT1y45 + COL10y50 and FIT1y45 + COL10y50-3X) had higher probability of being cost effective beyond the WTP of 20,000 € per QALYG. The result of the Monte Carlo simulation on the ICER is mentioned in the Supplementary material. See Supplementary Figures S2–S4 for detailed information.

## 4 Discussion

This research examined the impact of starting CRC screening at age 45 versus age 50. Our simulation results revealed that initiating a three times 10-yearly colonoscopy at 45 years or implementing an annual FIT strategy from ages 45 to 49 with a transition to colonoscopy at age 50 and 60 yielded favorable outcomes, including reduced colorectal cancer cases, prevented deaths, and increased quality-adjusted life-years compared to the current strategy. However, the magnitude of these health benefits depended on screening adherence.

Given the rising incidence of early onset CRC among younger individuals, commencing CRC screening at the age of 45 has emerged as a prospective strategy to address this emerging trend (4, 41). CRC screening and early detection can not only reduce CRC mortality but also can reduce cancer incidence by removal of precancerous lesions (7). Moreover, early detection and treatment of CRC can lead to improved survival rates and lower treatment costs compared to advanced-stage treatments, while also avoiding the need for invasive and costly interventions (7).

The latest US screening guidelines recommend the initiation of CRC screening at age 45 years, a reduction from the previous age of 50 years (20, 42, 43). This was partly due to the findings from an US modeling study in 2021 utilized three well-established CRC models (SimCRC, CRC-SPIN, and MISCAN), which supported the policy change (20). With perfect adherence, the outcomes consistently showed that adjusting the starting age for three times 10-yearly colonoscopy from 50 to 45 years can prevent an average of 3 CRC cases and 1 CRC-related death, while also leading to 2 colonoscopy complications and requiring 784 more colonoscopies per 1,000 individuals (20).

In our model, simulating the same strategies with perfect adherence resulted in 2 fewer CRC cases, 1 prevented death, 0.36 colonoscopy complications, and 282 additional colonoscopies. All these model findings concur that commencing CRC screening at age 45 yields substantial health benefits compared to starting at 50 years of age. But, making a direct comparison between the studies is intricate due to methodological variations, distinct model structures, and differing assumptions. The primary factor accounting for the divergence in colonoscopy requirement counts between the US models and the DECAS model may stem from distinct assumptions regarding test sensitivity, the integration of two pathways within our model, and variations in surveillance intervals for colonoscopy. See detailed comparison in Supplementary Table S4.

Among the strategies evaluated in this study, the colonoscopy-only approach, recognized as the gold standard test for its superior sensitivity and specificity, emerges as the most favorable strategy in terms of both effectiveness and cost-effectiveness. However, its resource-intensive nature, encompassing facilities and technicians, warrants consideration (20). To address whether the observed benefits resulted from early screening or a 3-time colonoscopy (COL), we conducted a sensitivity analysis. Our model outcomes show that adopting the COL strategy with colonoscopies at ages 45, 55, and 65 (COL10y45-3X) leads to a 15.15 CRC incidence and 5.53 CRC mortality. In contrast, the COL strategy with colonoscopies at ages 50, 60, and 70 (COL10y50-3X) results in a 17.25 CRC incidence and 6.53 CRC mortality (the latter details omitted in the main table). Compared to the scenario of no screening (57.92 CRC incidence and 27.05 mortality), initiating the 3-times 10-yearly colonoscopy 5 years earlier could potentially achieve an additional 4% reduction in both CRC incidence and mortality.

Evaluating the incremental number of colonoscopies required is pivotal for effective resource management. In contrast to the mixed (FIT + Colonoscopy) strategies, the colonoscopy-only approach mandates the highest incremental NNC, potentially tied to colonoscopy complications. On the other hand, increasing usage of non-invasive screening options like FIT is considered more user friendly, but false positives could lead to anxiety and unnecessary follow-up testing (20). Striking a balance between resource allocation, benefits, and potential harm remains imperative for informed decision making.

Furthermore, screening adherence plays a crucial role on the effectiveness of CRC screening intervention. In scenarios of perfect adherence, COL10y45-3X emerges as the preferred option with the lowest ICER compared to the current strategy. However, when considering imperfect adherence, both COL10y45-3X and FIT1y45 + COL10y50 strategies lie on the efficiency frontier and the ICERs of the screening strategies also changed prominently. The dynamic shift of the efficiency frontier due to screening adherence emphasizes its profound influence on the benefits of screening.

Much can be learned from some European CRC initiatives to improve screening adherence, such as those in the Netherlands and Basque country in Spain. Notably, their employment of mail-out FIT screening, advanced notifications, and reminder letters helped achieve participation rates ranged from 44 to 75% (22, 23, 26, 44). By integrating cost inputs for such approaches into Scenario 3 of our study and assuming high adherence rates, we unveil significant alterations in the screening effectiveness of each strategy. Improving adherence could require tailored approach, including proactive invitations and awareness campaigns. Reminders can be effective, but barriers to non-attendance may vary across countries (45).

### 4.1 Strength and limitation

The biggest strength of this study is that it is the first cost-effectiveness analysis of CRC screening initiation at 45 years of age instead of 50 years in the German context. No German studies have examined whether beginning CRC screening at age 45 can balance benefits and harm. Our study’s findings align with other screening recommendations and could serve as a basis for potential changes in Germany.

We also acknowledge several limitations in this study. Notably, the model input parameters, encompassing variables like test sensitivity, specificity, utility values, and cost inputs, are derived from diverse studies conducted in different countries. This variance in reference sources introduces potential uncertainties in the model output, as the applicability of these parameters to the specific context of the German population might differ.

While a perfect adherence scenario may not be achievable in reality, it represents the maximum potential effect of a specific population screening strategy for comparison. The scenario 2 assumed screening participation rates based on real rates until 2017 and increased rates observed in German RCTs, but it is unclear how real-world participation rates under the organized screening program behave. The potential rise in screening participation could amplify screening benefits. A repeated scenario analysis should follow the availability of post-organized CRC screening program participation rates for a more precise economic evaluation of the German program.

A direct comparison with other studies must be approached with caution due to differences in model structure, parameters, adherence scenarios, and other factors, the overall conclusions are generally consistent. It should be noted that this study only examined 4 alternative screening strategies involving FIT and colonoscopy, and did not include other recommended strategies such as annual to 3-yearly multi-target stool DNA test (mtsDNA) and 5-yearly computed tomographic colonography (CTC) as per other guidelines.

### 4.2 Future research direction

Further research and analysis are warranted to explore potential improvements in CRC screening strategies and adherence rates. The discrete event simulation model structure which is a variant of microsimulation models enables analysis by tumor location, stage and other features. However, this study does not explore these additional aspects. The DECAS model can be further used as a base platform to provide modeling evidence for various screening modalities or risk-stratified screening strategies (e.g., with a prior individual risks), either in the German context or other geographic regions with adaptation to the local CRC epidemiology.

## 5 Conclusion

This cost-effectiveness information can serve as a basis to inform future CRC screening policy-making to initiate CRC screening at 45 years of age in Germany. Our findings emphasize the importance of implementing CRC screening 5 years earlier than the current practice to achieve more significant health and economic benefits. However, other factors should also be considered in CRC screening policy-making, such as the clinical implications, the health care resources, the patient preferences, and the real-world adherence of the screening program.

## Data availability statement

The original contributions presented in the study are included in the article/Supplementary material, further inquiries can be directed to the corresponding authors.

## Author contributions

MWL: Conceptualization, Formal analysis, Investigation, Methodology, Project administration, Software, Validation, Visualization, Writing – original draft, Writing – review & editing. C-YC: Conceptualization, Data curation, Formal analysis, Investigation, Methodology, Software, Validation, Writing – review & editing. SC: Conceptualization, Methodology, Writing – review & editing. CS: Conceptualization, Methodology, Writing – review & editing. MS: Conceptualization, Funding acquisition, Methodology, Resources, Supervision, Writing – review & editing.

## Glossary

CEA: cost-effectiveness analysis
CEACs: cost-effectiveness acceptability curves
CI: confidence interval
COL: colonoscopy
CRC: colorectal cancer
CRC-SPIN: Colorectal cancer simulated population model for incidence and natural history
CTC: computed tomographic colonography
DECAS: Discrete event simulation model for the natural history of colorectal cancer from the adenoma and serrated neoplasia pathways
EQ-5D-3L: European quality of life 5 dimensions 3 level version tool
FIT: fecal immunochemical test
gFOBT: guaiac fecal occult blood test
ICER: incremental cost-effectiveness ratios
LYG: life-years gained
MISCAN: Microsimulation screening analysis – colorectal cancer model
mtsDNA: multi-target stool DNA test
NHB: net health benefit
NNC: number needed for colonoscopy
PSA: probabilistic sensitivity analyses
QALYG: quality-adjusted life-years gained
SHI: Statutory health insurance
SimCRC: Simulation model of colorectal cancer (Minnesota/MGH)
WTP: willingness-to-pay
