# Supplementary material for: Would initiating colorectal cancer screening from age of 45 be cost-effective in Germany? An individual-level simulation analysis
Source: Front Public Health. 2024 Feb 21;12:1307427. doi: 10.3389/fpubh.2024.1307427 (PMC10919152; doi:10.3389/fpubh.2024.1307427)
Supplement: Supplementary file 1 [file Data_Sheet_1.pdf]

Supplementary Material

Would Initiating Colorectal Cancer Screening from Age of 45 be Cost-Effective in Germany? An Individual-Level Simulation Analysis

Table of Contents **Page**

**Supplementary Figure 1. DECAS schematic model structure ……………………………………………………..**	1
**Supplementary Figure2. ICER scatter plot of (COL10y45-3X) compared to current screening strategy, perfect adherence scenario. (QALYG as benefits per 1000 individuals, 3% discount rate)**	2
**Supplementary Figure3. ICER scatter plot of (COL10y45-3X) compared to current screening strategy, current adherence scenario. (QALYG as benefits per 1000 individuals, 3% discount rate)**	3
**Supplementary Figure4. ICER scatter plot of (COL10y45-3X) compared to current screening strategy, high adherence scenario. (QALYG as benefits per 1000 individuals, 3% discount rate)**	4
**Supplementary Table 1. Summary of DECAS parameters and calibration results …………………………...**	5
**Supplementary Table 2. Assumptions of surveillance colonoscopy intervals after lesion removal**	6
**Supplementary Table 3. Modeled QALYs and lifetime costs of screening strategies per 1,000 40-years-old individuals with different discount rates.**	7
**Supplementary Table 4. Comparison of model outcomes with other US models ………………………………**	8
**Supplementary Table 5. Burden and harm of different screening strategies ...…………………..………....**	9
**References ………………………………………………………………………………………………………**	10

1. The base model for simulation

DECAS (Discrete Event simulation model for the natural history of colorectal cancer from the Adenoma and Serrated neoplasia pathways) is an individual-level CRC screening model that simulates the natural progression of CRC from the adenoma-carcinoma and serrated neoplasia pathways [1]. It simulates individual level CRC-related events from age 20 to 90 years or death, considering lesion initiation, pre-clinical stage, clinical cancers, and death based on state occupation. DECAS is constructed as a discrete event simulation in R software and it does not consider lesion regression. The model takes 21 transition-related parameters to generate event times and is calibrated using uniformly distributed priors into a Bayesian method for calibration. The R software version 4.0.4 was used for programming and calibration. See Supplementary Figure 1 and supplementary table 1. Further details on DECAS model structure, assumptions, calibration, and validation can be found in published work [1]. The natural history component includes assumptions about progression, while the screening component incorporates adherence, test performance, intervals, and test types.

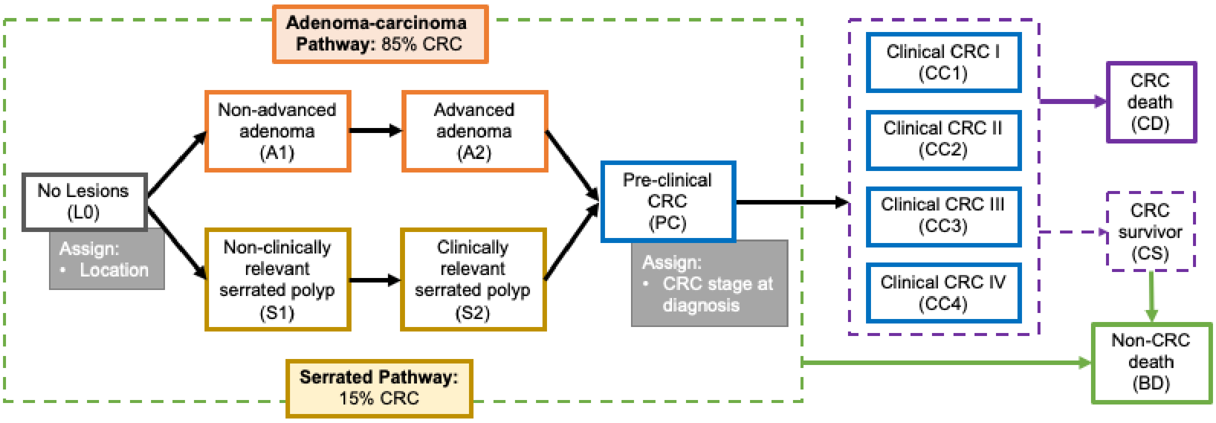

Supplementary Figure 1. DECAS schematic model structure.

2. Monte Carlo simulation on the ICER

In order to assess the cost-effectiveness of the most effective strategy (COL10y45-3X), a Monte Carlo simulation was conducted using 1000 random samples within the 95% confidence interval (CI) of the mean values of incremental cost and incremental quality adjusted life year gained (QALYG). These values were plotted on the cost-effectiveness plane. This approach take into account the uncertainty associated with the mean values within their respective confidence intervals. The ICER scatter plot showed that all 1000 random values of the ICERs lie in the right-upper quadrant in scenario 1 and scenario 2. In scenario 3, all the values lies in the right-lower quadrant showing its' the dominant to current strategy. See supplemental figure 2-4 below.

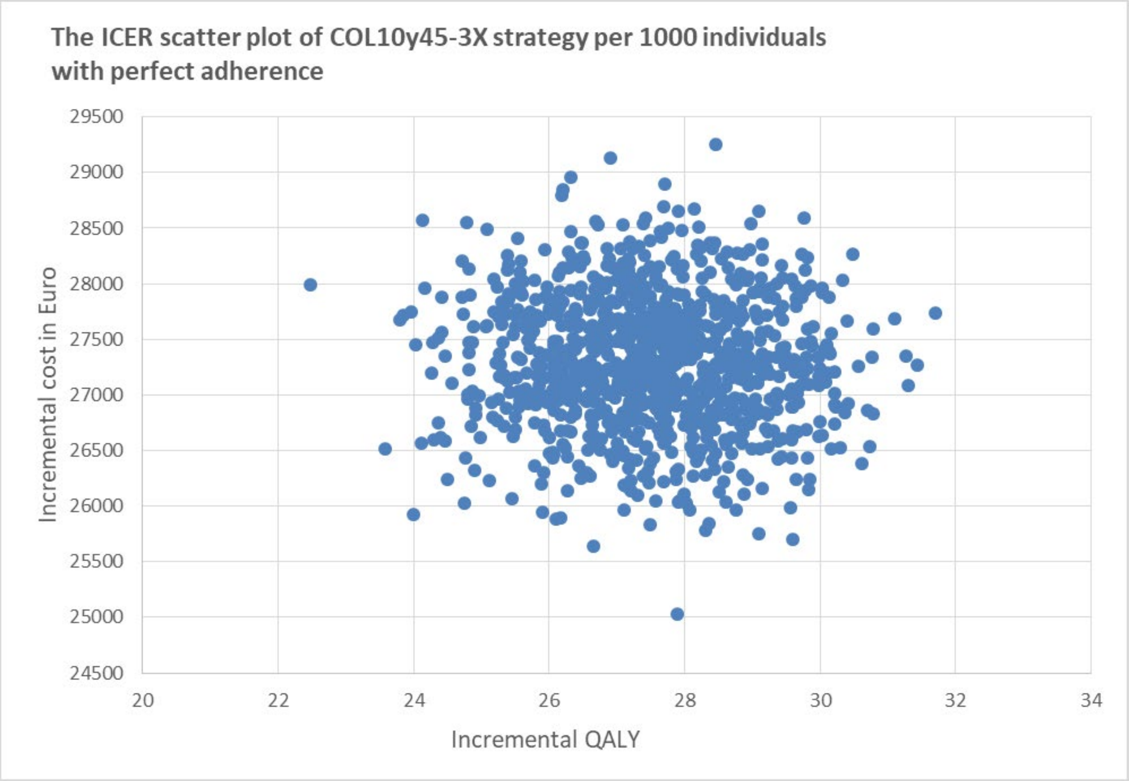

Supplementary Figure2. ICER scatter plot of (COL10y45-3X) compared to current screening strategy, perfect adherence scenario. (Cost and QALY per 1000 individuals, 3% discount rate).

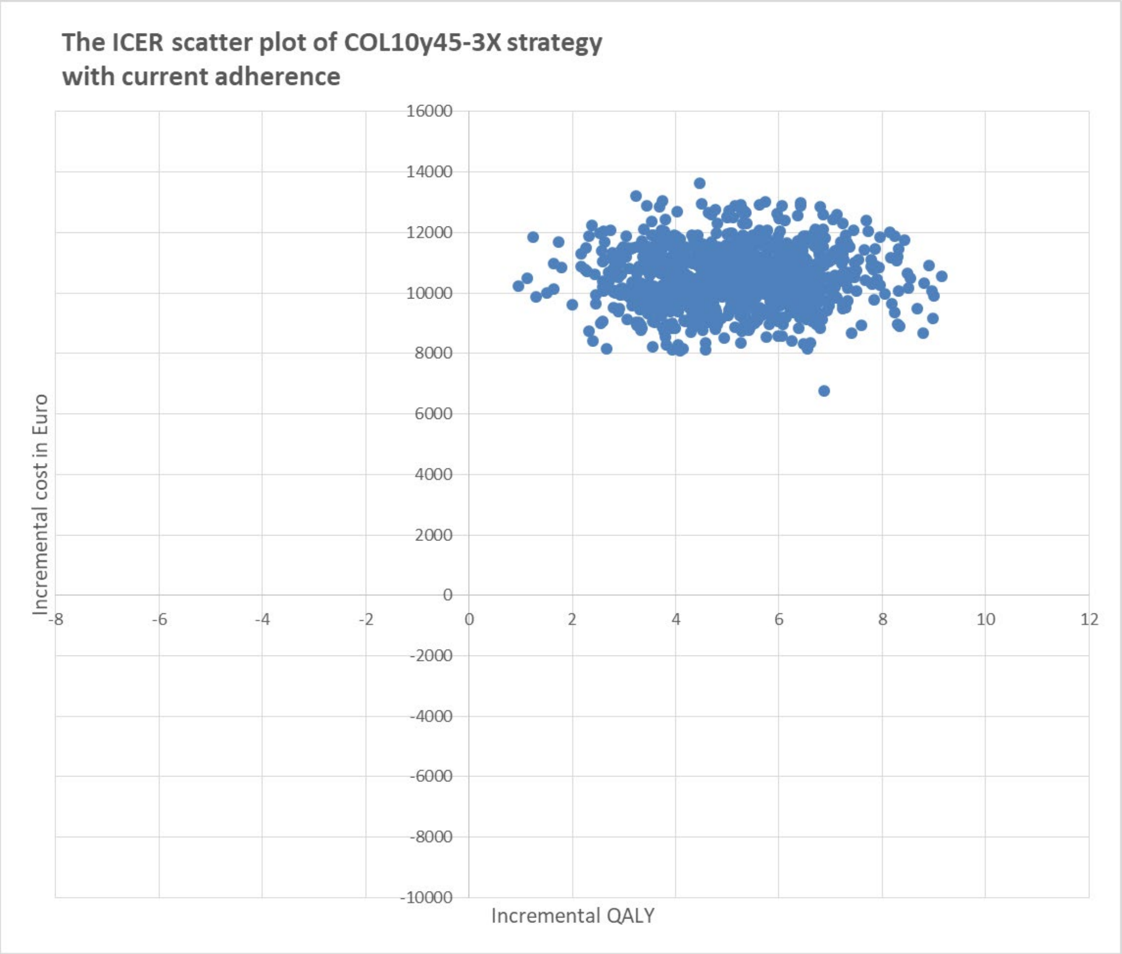

Supplementary Figure3. ICER scatter plot of (COL10y45-3X) compared to current screening strategy, current adherence scenario (Cost and QALY per 1000 individuals, 3% discount rate).

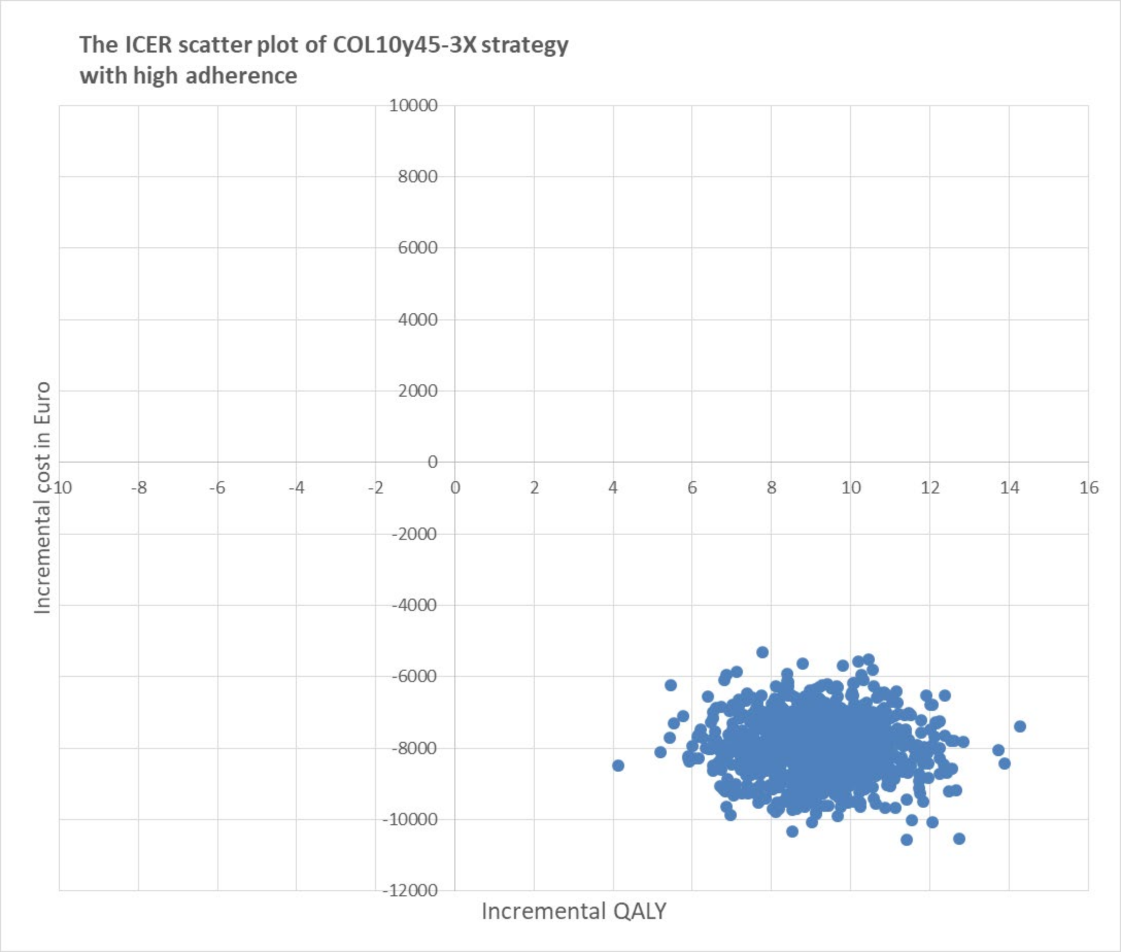

Supplementary Figure4. ICER scatter plot of (COL10y45-3X) compared to current screening strategy, high adherence scenario. (Cost and QALY per 1000 individuals, 3% discount rate).

3. Base Model Calibration results

Supplementary Table 1. Summary of DECAS parameters and calibration results

**Model parameters**	**Prior distribution**	**Posterior estimates Mean**	**95% CrI**	**Reference**
Adenoma				
Baseline log-risk, mean	~ U(-9, -4.6)	-7.240	(28.899, 24.970)	Assumption based on (Rutter[2]; Greuter [3]; Brenner[4])
Baseline log-risk, standard deviation	~ U(0.8, 5.4)	2.723	(1.693, 4.096)	
Sex effect	~ U(-0.65, 0)	-0.373	(-0.628,-20.078)	
Age effect, 20 ≤ age < 50 years	~ U(-0.06, 0.1)	0.023	(-0.035, 0.072)	
Age effect, 50 ≤ age < 70 years	~ U(-0.1, 0.15)	0.035	(-0.083, 0.137)	
Age effect, age ≥ 70 years	~ U(-0.1, 0.2)	0.036	(-0.091, 0.181)	
Serrated polyp				
Baseline log-risk, mean	~ U(-9.8, -5.4)	-8.648	(-9.744, -6.696)	Assumption based on (Greuter[3]; Lew[5])
Baseline log-risk, standard deviation	Same as in adenoma			
Sex effect	~ U(-0.65, 0.25)	-0.265	(-0.606, 0.142)	
Age effect, 20 ≤ age < 50 years	~ U(-0.12, 0.06)	0.018	(-0.033, 0.055)	
Age effect, 50 ≤ age < 70 years	~ U(-0.12, 0.15)	-0.005	(-0.111, 0.118)	
Age effect, age ≥ 70 years	~ U(-0.12, 0.2)	0.026	(-0.112, 0.185)	
Progression to the advanced stage of precancerous lesion (A1 to A2 or S1 to S2)				
Hazard of non-AA progressing to AA	~ U(0.002, 0.3)	0.004	(0.002, 0.012)	Assumption based on (Greuter[3]; Lew[5])
Hazard of non-crSP progressing to crSP	~ U(0.002, 0.6)	0.014	(0.005, 0.040)	
Adenoma				
Base risk of colonic lesion progressing to pre-clinical cancer, male at age 20 years	~ U(0.002, 0.3)	0.005	(0.002, 0.014)	Assumption based on (Rutter[2]; Brenner[4]; Greuter [3])
Location effect, rectum	~ U(2, 30)	5.871	(2.192, 23.252)	
Age effect, 50 ≤ age < 70 years	~ U(1, 5)	1.923	(1.040, 4.384)	
Age effect, age ≥ 70 years	~ U(1.2, 10)	4.004	(1.439, 9.395)	
Serrated polyp				
Base risk of colonic lesion progressing to pre-clinical cancer, male at age 20 years	~ U(0.002, 0.6)	0.004	(0.002, 0.008)	Assumption based on (Greuter [3]; Lew[5])
Location effect, rectum	~ U(4, 50)	18.897	(6.133, 47.341)	
Age effect, 50 ≤ age < 70 years	~ U(1, 5)	1.651	(1.022, 3.624)	
Age effect, age ≥ 70 years	~ U(1.2, 10)	3.761	(1.404, 9.123)	

Note: CI, credible interval; ~ U (a, b) denotes the uniform distribution bounded by (a, b); L0: no lesions; A1: non-advanced adenoma; S1: non-clinically relevant serrated polyp; A2: advanced adenoma; S2: clinically relevant serrated polyp; PC: pre-clinical cancer

4. Screening follow-up and surveillance management

Screening follow-up and surveillance management used in the DECAS model is summarized in Supplementary Table 2. In the context of FIT screening, participants who receive a negative result will continue to adhere to the screening schedule as outlined by the screening strategy. Conversely, if a participant tests positive for FIT, they will be referred to a FIT-positive colonoscopy for further examination. Should any lesions be identified during the colonoscopy procedure, they will be excised, sent for biopsy, and the participant will follow the surveillance colonoscopy schedule recommended by the German S3 guidelines for CRC [6]. In the event that the colonoscopy results are negative, the participant will undergo screening again in ten years.

Supplementary Table 2. Assumptions of surveillance colonoscopy intervals after lesion removal

Colonoscopy findings	DECAS surveillance interval
1-2 small tubular adenomas (<10 mm)	7.5 years[1]
3-4 adenomas OR<br>≥1 adenoma ≥10 mm or villous or high-grade neoplasia	3 years
≥ 5 adenomas	1.5 year[2]
Serrated lesions	Same as adenomas[3]

Note: (1) German S3 guidelines recommends 5-10 years – a mid-value was used.
(2) German S3 guidelines recommends <3 years – a mid-value was used.
(3) When counting the lesions in order to follow the recommended surveillance interval, adenomas and serrated polyps were counted together in DECAS.

5. Screening benefit with different discount rates

Supplementary Table 3. Modeled QALYs and lifetime costs of screening strategies per 1,000 40-years-old individuals with different discount rates.

Strategy	QALYs (0%)	dQALYs(3%)	Cost‡ (0%)	dCost‡ (3%)
No Screening	33306.83	19107.18	2626781.68	1,084,554
mCOL50/fFIT50+COL55*	33504.64	19177.80	1374787.08	754,393
FIT1y45+COL10y50	33524.26	19185.90	1394489.85	793,408
FIT1y45+COL10y50-3X	33523.47	19185.15	1458042.46	826,152
FIT2y45	33468.31	19156.33	1779982.98	847,507
COL10y45-3X	33549.97	19205.36	1327710.68	782,753
mCOL50/fFIT50+COL55*	33387.43	19134.57	2089260.02	895,412
FIT1y45+COL10y50	33390.09	19136.28	2080000.94	896,663
FIT1y45+COL10y50-3X	33392.91	19137.16	2080685.85	901,810
FIT2y45	33357.43	19121.85	2288299.21	948,385
COL10y45-3X	33394.93	19139.77	2092704.06	906,330
mCOL50/fFIT50+COL55*	33420.35	19145.96	1926740.70	883,574
FIT1y45+COL10y50	33444.34	19156.37	1906580.76	914,639
FIT1y45+COL10y50-3X	33444.84	19156.05	1916206.90	925,344
FIT2y45	33412.24	19137.78	2257748.24	1,032,285
COL10y45-3X	33430.72	19155.10	1910283.27	875,290

Note: * Current strategy, ‡ Cost in Euro, COL = colonoscopy; FIT = fecal immunochemical test; dQALY = discounted quality-adjusted life-years, dCost = discounted lifetime cost
(1) The quality-adjusted life-years and costs were discounted with 0% and 3% annual rates.
(2) Results are presented as mean values of 1000 simulation of an age Cohort of 100000 individuals (reported per 1000 population).

6. Comparison of model outcomes with other models

In 2021, a study conducted by the United States Preventive Services Task Force utilized three well-established CRC models (SimCRC, CRC-SPIN, and MISCAN) analyzed the effect of initiating a three times 10 yearly colonoscopy at the age of 45 years instead of 50 [7]. A comparison is made with US models' outcome and the DECAS's outcome.

Supplementary Table 4. Comparison of model outcomes with other US models

Model outcome	SimCRC	CRC-SPIN	MISCAN	Average value of US models	DECAS model
Additional life-years gained	34	32	16	27	14
Additional CRC cases averted	4	3	2	3	2
Additional CRC cases averted	2	1	1	1	1
Additional Colonoscopies complications	2	2	2	2	0.36
Additional Colonoscopies	798	800	756	784	282

Note: Results are presented as mean values from lifetime simulation of 1000 40-years-old individuals for initiating 3 times screening colonoscopy at 45 years instead of 50.

7. Burden and Harms

In general, strategies that had shorter screening intervals, an earlier starting age, and more colonoscopy were associated with higher resource utilization and burdens. In the perfect screening scenario (Scenario 1), FIT-only screening strategy had the highest number of FIT consumption, as expected and COL-only strategy demanded for the highest number of colonoscopy. The highest utilization of colonoscopies in perfect adherence scenarios ranged from 991 to 3,240 colonoscopies per 1,000 individuals.

Major bleeding and perforation events requiring hospitalization were deemed as complications of colonoscopy. The estimated occurrence of these complications was 4 cases per 10,000 colonoscopies based on the 2018 annual report of the program[8]. Other studies conducted in Germany from 2001-2008 and 2010-2013 reported similar complication rates of 17 per 10,000 colonoscopies[9],[10]. A meta-analysis of international data reported rates ranging from 1 to 3 per 10,000 colonoscopies, while the USPSTF systematic review estimated a rate of 3.3 per 10,000 colonoscopies[11]. For our analysis, a complication rate of 4 cases per 10,000 colonoscopies was assumed, with a sensitivity analysis range of 2 to 24 per 10,000 colonoscopies. See the Supplementary Table 5 for the detailed result.

Supplementary Table 5. Burden and harm of different screening strategies

Strategy	Burden and Harms		
	FIT kit required	COL required	COL complication
**No screening**	--	--	--
**Scenario 1 (Perfect adherence)**			
mCOL50/fFIT50+COL55	1,967 (1,863-2,076)	2,354 (2,070-2,687)	3.06 (0.59-5.63)
FIT1y45+ COL10y50	3,856 (3,645-4,065)	2,483 (2,168-2,828)	3.24 (0.62-5.99)
FIT1y45+COL10y50·3X	3,856 (3,644-4,064)	3,007 (2,644-3,411)	3.91 (0.76-7.11)
FIT2y45	9,782 (9,076-10,531)	991 (737-1,280)	1.29 (0.25-2.55)
COL10y45·3X	-	3,240 (2,825-3,700)	4.21 (0.83-7.76)
**Scenario 2 (current Program)**			
mCOL50/fFIT50+COL55	516 (493-540)	525 (481-575)	0.69 (0.13-1.29)
FIT1y45+ COL10y50	664 (634-693)	549 (497-609)	0.71 (0.14-1.35)
FIT1y45+COL10y50·3X	664 (633-694)	701 (640-768)	0.91 (0.17-1.69)
FIT2y45	2,173 (2,042-2,308)	114 (86-144)	0.15 (0.02-0.31)
COL10y45·3X	-	722 (658-793)	0.94 (0.17-1.76)
**Scenario 3 (High adherence)**			
mCOL50/fFIT50+COL55	1,509 (1,436-1,587)	979(891-1,082)	1.27(0.26-2.38)
FIT1y45+ COL10y50	2,884 (2,739-3,028)	1,090(976-1,222)	1.41(0.27-2.64)
FIT1y45+COL10y50·3X	2,884 (2,739-3,027)	1,351(1,229-1,496)	1.76(0.33-3.29)
FIT2y45	7,531 (7,060-8,032)	539(4,10-679)	0.7(0.12-1.4)
COL10y45·3X	-	1,290(1,178-1,415)	1.67(0.31-3.1)

Note: FIT= fecal immunochemical test, COL= colonoscopy,
The results are reported as mean values with 95% confidence intervals based on 1000 simulations.
The reported values represent the mean outcomes per 1000 population in an age cohort of 100,000 individuals.
These values represent the absolute number of required resources and burdens for each strategy, without comparing them to the current strategy.

References

1. Cheng C-Y, Calderazzo S, Schramm C, Schlander M. Modeling the Natural History and Screening Effects of Colorectal Cancer Using Both Adenoma and Serrated Neoplasia Pathways: The Development, Calibration, and Validation of a Discrete Event Simulation Model. MDM policy & practice. 2023;8(1):238146832211457.
2. Rutter CM, Miglioretti DL, Savarino JE. Bayesian Calibration of Microsimulation Models. Journal of the American Statistical Association. 2009;104(488):1338-50.
3. Greuter MJE, Xu X-M, Lew J-B, Dekker E, Kuipers EJ, Canfell K, et al. Modeling the Adenoma and Serrated Pathway to Colorectal CAncer (ASCCA). Risk analysis. 2014;34(5):889-910.
4. Brenner H, Altenhofen L, Stock C, Hoffmeister M. Incidence of colorectal adenomas: birth cohort analysis among 4.3 million participants of screening colonoscopy. Cancer epidemiology, biomarkers & prevention. 2014;23(9):1920-7.
5. Lew J-B, St John DJB, Xu X-M, Greuter MJE, Caruana M, Cenin DR, et al. Long-term evaluation of benefits, harms, and cost-effectiveness of the National Bowel Cancer Screening Program in Australia: a modelling study. The Lancet Public health. 2017;2(7):e331-e40.
6. German Guideline Program In Oncology, GGPO. Evidenced-based Guideline for Colorectal Cancer 2019 [Available from: https://www.leitlinienprogramm-onkologie.de/leitlinien/kolorektales-karzinom/.
7. Knudsen AB, Rutter CM, Peterse EFP, Lietz AP, Seguin CL, Meester RGS, et al. Colorectal Cancer Screening: An Updated Modeling Study for the US Preventive Services Task Force. JAMA : the journal of the American Medical Association. 2021;325(19):1998-2011.
8. Screening colonoscopy Annual Report 2018, The Central Institute for Statutory Health Insurance Physician Care in the Federal Republic of Germany (Zi). Zentralinstitut fur die kassenarztliche Versorgung in Deutschland, Cologne.; 2020. Contract No.: 16.04.2023.
9. Zwink N, Holleczek B, Stegmaier C, Hoffmeister M, Brenner H. Complication Rates in Colonoscopy Screening for Cancer. Deutsches Ärzteblatt international. 2017;114(18):321-7.
10. Stock CP, Ihle PMD, Sieg AMD, Schubert IP, Hoffmeister MP, Brenner HMDMPH. Adverse events requiring hospitalization within 30 days after outpatient screening and nonscreening colonoscopies. Gastrointestinal endoscopy. 2013;77(3):419-29.
11. Jodal HC, Helsingen LM, Anderson JC, Lytvyn L, Vandvik PO, Emilsson L. Colorectal cancer screening with faecal testing, sigmoidoscopy or colonoscopy: a systematic review and network meta-analysis. BMJ open. 2019;9(10):e032773-e.
